# Epigenetic Markers and Microbiota/Metabolite-Induced Epigenetic Modifications in the Pathogenesis of Obesity, Metabolic Syndrome, Type 2 Diabetes, and Non-alcoholic Fatty Liver Disease

**DOI:** 10.1007/s11892-019-1151-4

**Published:** 2019-05-01

**Authors:** Daniela Stols-Gonçalves, Luca Schiliró Tristão, Peter Henneman, Max Nieuwdorp

**Affiliations:** 1 Department of Vascular Medicine, Amsterdam UMC, Location AMC, Meibergdreef 9 (Room A01-112), 1105 AZ Amsterdam, The Netherlands; 2 Faculdade de Ciências Médicas de Santos (UNILUS), R. Oswaldo Cruz, 179, Boqueirão, Santos, SP 11025-020 Brazil; 3 Department of Clinical Genetics, Amsterdam UMC, Location AMC, Meibergdreef 9 (Room A01-112), 1105 AZ Amsterdam, The Netherlands

**Keywords:** Epigenetics, Microbiota, T2D, Insulin resistance, Adipose tissue, Metabolic syndrome, Obesity, NAFLD

## Abstract

**Purpose of Review:**

The metabolic syndrome is a pathological state in which one of the key components is insulin resistance. A wide spectrum of body compartments is involved in its pathophysiology. Genetic and environmental factors such as diet and physical activity are both related to its etiology. Reversible modulation of gene expression without altering the DNA sequence, known as epigenetic modifications, has been shown to drive this complex metabolic cluster of conditions. Here, we aim to examine some of the recent research of specific epigenetically mediated mechanisms and microbiota-induced epigenetic modifications on the development of adipose tissue and obesity, β-cell dysfunction and diabetes, and hepatocytes and non-alcoholic fatty disease.

**Recent Findings:**

DNA methylation patterns and histone modifications have been identified in this context; the integrated analysis of genome, epigenome, and transcriptome is likely to expand our knowledge of epigenetics in health and disease. Epigenetic modifications induced by diet-related microbiota or metabolites possibly contribute to the insulin-resistant state.

**Summary:**

The identification of epigenetic signatures on diabetes and obesity may give us the possibility of developing new interventions, prevention measures, and follow-up strategies.

## Introduction

### Insulin Resistance, the Metabolic Syndrome, Type 2 Diabetes, and Non-alcoholic Fatty Disease

The underlying pathophysiology of the metabolic syndrome remains to be completely elucidated. This syndrome has been called by at least eight different names like metabolic trisyndrome, syndrome X, deadly quartet, or insulin resistance syndrome and has included different features like gout, hyperlipidemia, impaired glucose tolerance, type 2 diabetes (T2D), hypertension, and central adiposity [1]. The Indian surgeon Susruta was one of the first known physicians who in 600 BC linked obesity and diabetes and who used to prescribe exercise to minimize its consequences [2]. Hippocrates, who lived 460–370 BC, wrote that “corpulence is not only a disease itself but the harbinger of others” [3].

A current widely accepted definition of metabolic syndrome proposed by a consensus from the International Diabetes Federation and the American Heart Association/National Heart, Lung, and Blood Institute is a constellation of three or more from five risk factors: abdominal obesity, high triglycerides, low level of high-density lipoprotein cholesterol, high blood pressure, and elevated fasting blood glucose [4].

Insulin resistance (IR) is believed to be a common factor that is strongly correlated to most components of the metabolic syndrome [5]. It is a state in which the target tissues like skeletal muscle, adipose tissue, and the liver respond inadequately to insulin [6].

### Points of Attention Reading Epigenetic Studies

**Fig. 1:**
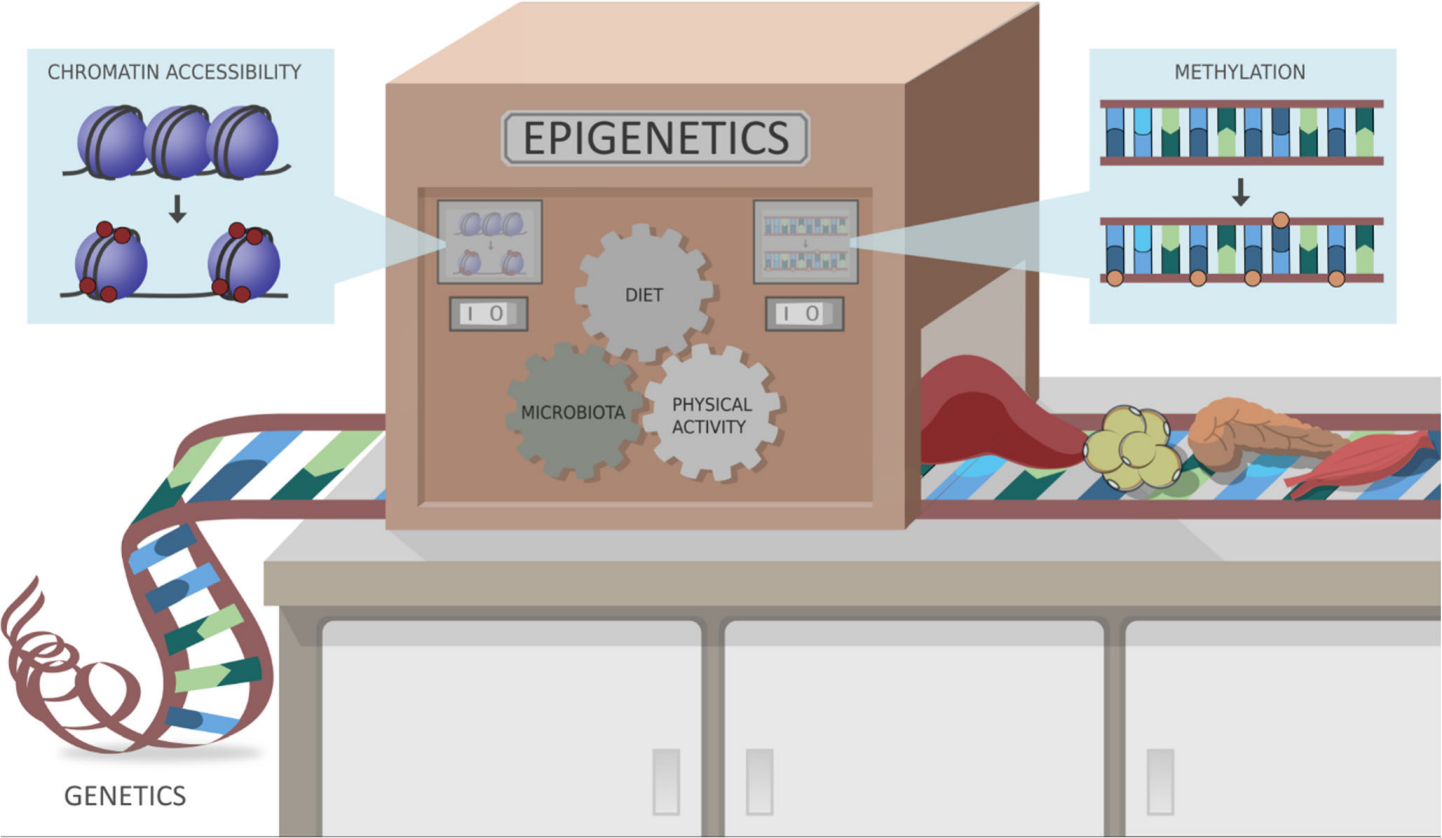
Epigenetic is the collective of heritable changes in phenotype that occur independent of the primary DNA sequence, altering the gene expression. Epigenetic modifications can alter chromatin accessibility, through for example acetylation and methylation of DNA and histones. These modifications can be induced by different agents such as diet, microbiota, metabolites, and physical activity

The field of epigenetics studies the contribution of endogenous and exogenous factors (diet, gut microbiota, environment, and medication) on human phenotype changes that do not involve mutation in the DNA sequence, but rather affect transcription (e.g., via acetylation or methylation of histone proteins, or methylation of DNA itself). In this review, we will therefore try to offer a glimpse of the current knowledge of epigenetic factors and their role on the insulin-resistant metabolic state. We will also review gut microbiota and their derived metabolites and their effect on epigenetic markers (Fig. 1).

As mentioned, the field of epigenetics studies the modulation of gene expression and function that occurs without altering the DNA sequence. Epigenetic modifications can be caused by alterations in DNA methylation, modifications to histone proteins, remodeling of chromatin, and RNA-based mechanisms, such as non-coding RNAs [7]. There is some controversy in considering all of these as epigenetic marks, as some believe that a property needed to define a true epigenetic mark is to be able to carry information through cell division, while some argue that some histone modifications and high-order chromatin structure frequently lack this property and therefore their mention as epigenetic elements should be qualified [8]. Modifications of histone tails are, however, considered as critical mechanisms in the activation and repression of gene transcription [9]. The epigenome is the epigenetic information in a cell, comprising DNA methylation, post-translational modifications of histones, and higher-order chromatin structure [10].

In this regard, an important question is how epigenetic information is stored and inherited [11], also known as “epigenetic memory.” D’Urso and Brickner refer to “epigenetic memory” as the propagation of a change in gene expression that can possibly happen through different mechanisms: cellular memory, transcriptional memory, and transgenerational memory [12].

When analyzing epigenetic research, one should not forget that there is cross-talk between these modifications. This combination will probably be determinant of the overall modulation of transcriptional outcome, by activating or repressing gene expression [13]. For example, recently it has been demonstrated that the trimethylation of lysine 36 in histone H3 (by convention, H3K36me3) stimulates acetylation of lysine 16 in histone 4 (H4K16ac) [14]. Some histone modifications are also associated with DNA methylation at CpG islands, CpG-rich regions that are present in the promoters of many genes [9]. A simultaneous analysis of the genome, epigenome, and transcriptome (the set of genes expressed in a given state) [15] enriches the chances of elucidating how an epigenetic marker might influence the expression of a specific gene. When we consider DNA methylation, it is crucial to note that its effect on gene expression varies according to different genomic contexts, such as transcriptional start sites with or without CpG islands, in gene bodies, at regulatory elements, and at repeat sequences, leading to either up- or downregulation of gene expression [16].

Another point of attention while reading epigenetic studies is the question of the use of surrogate tissue, a situation in which the health or condition of an inaccessible “target” tissue (e.g., liver or β-cells) is determined by analyzing an accessible or “surrogate” tissue such as peripheral blood leukocytes [17]. We will highlight this in this paper when we discuss some recent studies that have analyzed genome-wide or specific gene DNA methylation and histone modification in relation to bacterial metabolite-induced epigenetic marks.

## Obesity and Epigenetic Modulation

With a rising prevalence especially in Western world countries, obesity results from an interplay between genetic susceptibility, diet, epigenetics, metagenomics, and the environment [18]. To investigate whether blood can be used as a surrogate tissue for adipose tissue, one recent population-based epigenome-wide study collected data, from adipose tissue from the upper outer quadrant of the buttock and blood, in 143 healthy subjects, to try to extend knowledge regarding the similarity between adipose tissue and blood. In general, subcutaneous adipose tissue samples were hypomethylated compared with blood samples. Genes were divided into two groups: concordant genes (with smaller gene methylation variation between the two groups) and discordant (with a higher variation). Gene ontology analysis was performed to elucidate their role in obesity and showed that whereas concordant genes (identified as mainly responsible for maintaining basic cellular functions) had constant gene expression pattern across tissues, discordant genes (critical for tissue-specific biological functions) had distinct epigenetic and transcription patterns [19]. In another study, DNA methylation from peripheral blood leukocytes and adipocytes derived from the upper outer quadrant of the buttock was performed in 106 middle-aged men and women. Although there was no significant association between blood leukocyte DNA methylation and adiposity, adipose tissue DNA methylation profiles were associated with measures of adiposity, including centrally located fat, body fat distribution, and body mass index, reinforcing the concept that tissue-specific DNA methylation patterns influence adipose tissue regulation in human obesity [20].

In this regard, mammals possess two types of adipose tissue: white adipose tissue (WAT) and brown adipose tissue (BAT) [21]. WAT functions like energy stores while BAT is a major site of thermogenesis, thus regulating body temperature and energy expenditure. Beige (brite) adipocytes are the brown adipocytes appearing in WAT [22]. WAT and BAT are contained in the subcutaneous and visceral compartments and are capable of reciprocal reversible transdifferentiation according to physiological needs: the need for thermogenesis induces browning and positive energy balance induces whitening [23]. A study in mice who were exposed to cold has confirmed the notion that rodent inguinal WAT is the adipose depot most prone to browning. An increased transient level of zinc finger (Zic1 mRNA) expression was found during the early browning process. Although overall DNA methylation did not appear to be related to this gene expression, a repressive H3K9me histone mark was found as a possible epigenetic feature involved in the early stages of this white-to-brown differentiation (Table 1) [21]. It is thought that DNA methylation in human subcutaneous adipose tissue and omental visceral adipose tissue from non-obese vs. obese individuals has depot-specific differences. Keller et al. have indeed identified those differences in new candidate genes and in previously known obesity-related genes. Some of the differentially methylated genes were *HAND2*, *HOXC6*, *PPARG*, *SORBS2*, *CD36*, and *CLDN1*. Specifically, the *PPARG* promoter region was differently methylated in omental visceral adipose tissue with decreased gene expression when compared with subcutaneous adipose tissue. *PPARG* is a key regulator of adipogenesis and adipocyte differentiation (Table 1) [24].

**Table 1:** Examples of epigenetic modifications and microbiome-related modifications in obesity, type 2 diabetes, and NAFLD

	Obesity	Type 2 diabetes	NAFLD
Epigenetics	- H3K9me in WAT - Differently methylated obesity-related genes: *HAND2*, *HOXC6*, *PPARG*, *SORBS2*, *CD36*, *CLDN1* - Differently methylated *PPARG* promoter region in omental visceral compared with subcutaneous adipose tissue	- Differently methylated genes: *TCF7L2*, *THADA*, *KCNQ1*, *FTO*, *IRS1* - H3K27me3 modification of myocytes - Metformin indirectly induced hypermethylation of tumor-promoting pathway genes and inhibits cell proliferation	- Hypermethylation *PNPLA3* - Differently methylated MT-DN6 gene
Microbiome	Butyrate inhibition of HDAC3 and HDAC1 reduced methylation in obesity-associated genes *FTO* and *MC4R* upon probiotic supplementation	Butyrate inhibition of HDAC differently methylated palmitate-treated human islets in genes *TCF7L2*, *GLIS3*, *HNF1B*, and *SLC30A8*	Bacterial dysbiosis leading to increased permeability, endotoxemia, and increased pro-inflammatory cytokines

*H3K9me*, methylation of lysine 9 on histone 3; *WAT*, white adipose tissue; *HDAC*, histone deacetylase; *HDAC3*, histone deacetylase 3; *HDAC1*, histone deacetylase 1; *MT-ND6*, mitochondrially encoded NADH dehydrogenase 6; *H3K27me3*, trimethylation of lysine 27 on histone 3; *PNPLA3*, patatin-like phospholipase domain containing 3

## Gut Microbiota–Derived Metabolites, Epigenetics, and Obesity

The gut microbiota refers to the microbes that collectively inhabit the intestinal tract of an animal organism, and the microbiome is the collection of all genomes from microbes in an ecosystem [25]. Sender et al. have elegantly estimated the total number of bacteria in the colon, in a 70-kg “reference male,” to be approximately 3.8 × 10^13^ [26]. The human gut microbiota is mainly composed of four phyla: *Firmicutes*, *Bacteroidetes*, *Actinobacteria*, and *Proteobacteria* [27]. However, besides bacteria, feces also contain viruses, fungi, and human intestinal epithelial cells, and it is thought that all of these components drive overall microbiome composition [28]. In this regard, metabolites derived from gut microbiota or consumed foods may influence epigenetic mechanisms contributing to human disease including obesity and IR [29]. Butyrate, for example, is a short-chain fatty acid (SCFA) that is the primary fuel source for colon enterocytes, thus influencing gut homeostasis. This SCFA is derived from microbiota and originates from the fermentation of dietary fiber, with *Roseburia*, *Eubacterium hallii*, and *Faecalibacterium prauznitzii* among the most common bacterial strains producing this SCFA [30]. Butyrate also inhibits histone deacetylase 3 (HDAC3), an enzyme that modulates histone acetylation in intestinal epithelial cells thereby affecting metabolic control. As expected, wild-type C57BL6 mice fed with a high-fat diet gained weight and became obese, while HDAC3 knockout mice did not develop obesity despite being on the same high-fat diet, and had less liver fat and smaller adipocytes. In addition, butyrate supplementation inhibits histone deacetylase 1 (HDAC1), leading to downregulation of the inflammatory pathway in rodents with colitis (Table 1). Inhibition of HDAC1 downregulates the IL-6/STAT3/IL-17 pathway and promotes Foxp3 expression leading to an altered Th17/Treg ratio, causing an anti-inflammatory effect [31]. As diet-induced obese mice had significant weight reduction after oral SCFA butyrate administration [32], taken together, these data underscore the potential role of epigenetic modifications in developing an obese phenotype. The question however remains whether beneficial effects on metabolism are also seen in human obesity.

Recently, we have reported a comparative study of oral butyrate supplementation in lean individuals and in subjects with the metabolic syndrome. Intriguingly, oral butyrate treatment improved metabolism in lean individuals, yet we observed no effect in participants with the metabolic syndrome. We had to use the maximum allowed dose of 4 g/day, a limit based on previous literature [33]. This possible “sub-therapeutic dose” not adjusted by weight might explain why a positive effect on insulin sensitivity was only seen in lean subjects. However, it may also be possible that intestinally produced SCFAs are handled differently in the obese insulin-resistant state, to regulate glucose and lipid metabolism [34]. In support of this hypothesis, we have previously shown the beneficial effect on glucose metabolism of fecal microbiota transplantation from lean donors to subjects with the metabolic syndrome [35]*.* As these changes were associated with alterations in fecal SCFA levels, it is possible that epigenetic mechanisms underlie these beneficial effects, a hypothesis currently under investigation.

Another example of the microbiome and epigenetic interactions was shown by Vähämiko et al., who showed that DNA methylation was reduced in the promoters of the obesity-associated genes *FTO* and *MC4R* in women who had probiotic supplementation with *Lactobacillus rhamnosus* GG and *Bifidobacterium lactis* Bb12 and their children during pregnancy (Table 1) [36]. In this regard, early-life (intra-uterine) exposure to these gut microbiota and their diet-derived metabolites might influence the development of the infant’s microbiome post-partum and the long-term regulation of metabolism [37]. Future research focusing on the epigenome of pregnant women and their children and its relationship with the composition of amniotic fluid is needed.

## Role of Microbiota-Derived Metabolite Epigenetic Regulation in NAFLD-NASH

Regarding IR and the liver, an association has been observed between IR and non-alcoholic fatty liver disease (NAFLD). NAFLD refers to a wide spectrum of liver damage, ranging from simple steatosis to steatohepatitis, advanced fibrosis, and cirrhosis [38]. NAFLD (including non-alcoholic fatty liver “NAFL” and subsequent non-alcoholic steatohepatitis “NASH”) is regarded as a common complication of IR and T2D in obese subjects [39]. Whether IR precedes or is a consequence of NAFLD is however still a matter of debate [40].

Genetic variation has also been associated with the presence of NAFLD; for example, variation in the patatin-like phospholipase domain containing 3 (*PNPLA3*) gene is linked to differences in hepatic fat content and susceptibility to NAFLD [41]. A variant of the *TM6SF2* gene has also been linked to increased liver fat [42]. Other gene variants have been linked to NAFLD like *LYPLAL1*, *GCKR*, *APOB*, *MTTP*, *LPIN1*, *DOS2*, *UCP2*, *ENPP1*, *IRS1*, *IL28B*, *MERTK*, and irisin [7]*.* Another recent finding was a reduced risk from progression from steatosis to steatohepatitis in patients with a loss-of-function variant in the *HSD17B13* gene [43]. Interestingly, DNA methylation seems to regulate *PNPLA3* gene expression. For example, the regulatory region of *PNPLA3* was hypermethylated in human liver biopsy samples, with *PNPLA3* mRNA levels being lower in patients with advanced NAFLD compared with those with mild NAFLD, demonstrating a relationship with the severity of the disease (Table 1) [44].

Moreover, genome-wide DNA methylation analysis study using peripheral blood leukocytes has identified six differentially methylated CpG sites in patients with NAFLD (simple steatosis and NASH) compared with healthy controls. Serum liver enzymes and plasma cholesterol levels were directly correlated with the level of DNA methylation as well as the presence of simple steatosis or NASH [45].

As liver mitochondrial dysfunction seems to play an important role in disease progression, mitochondrial DNA methylation was evaluated in liver biopsies of 23 subjects with simple steatosis, 22 subjects with biopsy-proven NASH, and 18 subjects with near-normal liver histology. An association with histological severity was found in hepatic DNA methylation and the transcriptional activity of the mitochondrially encoded NADH dehydrogenase 6 (MT-ND6), a key protein involved in mitochondrial function (Table 1) [46]. Subsequently, another study showed that genes encoding liver proteins were also differently methylated in patients with advanced NAFLD (fibrosis stage 3–4) compared with those with mild (fibrosis stages 0–2) NAFLD [47]. Moreover, De Mello et al. have shown that differential liver DNA methylation was not only associated with NASH compared with normal liver and simple steatosis but also particularly correlated with fasting plasma insulin, suggesting a relationship with the expression of genes involved in hepatic insulin signaling [48].

Different mechanisms have been studied regarding the potential etiological role of the microbiome and of DNA methylation in NAFLD. Bacterial dysbiosis contributes to lowering the expression of tight junction proteins in the intestinal epithelium, leading to increased permeability, bacterial translocation, and endotoxemia. These endotoxins cause an increase in pro-inflammatory cytokines, IR, and hepatic lipid accumulation (Table 1) [49]. In support of the potential association between altered gut microbiota composition and liver inflammation in NAFLD, *Faecalibacterium prauznitzii* was inversely correlated with CD45+ and CD163+ in NAFLD, whereas *Prevotella* was negatively correlated with CD20+ [49].

## Epigenetics, Microbiota, and T2DM

Since the first genome-wide association study (GWAS) in T2D in 2007, over 400 genome-wide significant loci have been identified. Most (but not all) are also associated with impaired β-cell function. However, these genetic variants explain only ~ 20% of T2D heritability, in part possibly due to the inclusion of participants of mostly European descent in these studies. Future research on ethnic-specific variants, copy number variants such as small deletions or insertions, and rare functional variants of strong effects may increase our understanding of T2D heritability using large-scale biobanks in diverse populations [6, 50, 51].

Indeed, the association between DNA methylation and T2D has been reported repeatedly over the last years. For example, Dayeh et al. performed a genome-wide DNA methylation analysis of human pancreatic islets harvested from donors with and without T2D. The authors selected differentially methylated genes for functional analysis of insulin and glucagon in vitro using clonal β and α cells in which those genes were silenced or overexpressed. They identified 853 unique genes with differential DNA methylation, which included 17 genes previously identified in GWAS as affecting the risk of T2D, such as *TCF7L2*, *THADA*, *KCNQ1*, *FTO*, and *IRS1* (Table 1). This reinforces the idea that genetic and epigenetic mechanisms may interact to affect pancreatic β-cell function, development of IR, and T2D [52]. Furthermore, in 2017, Volkov et al. expanded their first study, performing their analysis with whole-genome bisulfite sequencing, a method that offers a more complete picture of the human islet methylome in T2D. Interestingly, the authors also integrated their findings of differentially methylated regions with previously published maps of histone modifications and enhancer regions in human islets and found that demethylation regions within the genome were associated with specific histone markers [53••]. This reinforces the importance of cross-talk between genome, transcriptome, and epigenome in islets. Varshney et al. found genetic variants that are linked to disruption of regulatory factor X binding, a family of transcription factors involved in glucose metabolism [54]. Because skeletal muscle is a key regulator of insulin sensitivity [55], it has also been examined in epigenetic research. The notion of a muscular epigenetic memory has been proposed, defined as “the capacity of skeletal muscle to respond differently to environmental stimuli in an adaptive or maladaptive manner if the stimuli have been previously encountered” [56]. Trying to identify intrinsic myocyte properties in T2D a gene-set analysis of histone modifications was performed in muscle biopsies taken from 24 subjects, divided into four groups (normal glucose tolerant or insulin resistant/T2D, both in obese and non-obese). In all groups, a significant histone modification was found in H3K27me3 (trimethylation of lysine 27 on histone 3), with a subsequent downregulation of genes involved in muscle function and upregulation of genes involved in inflammation in T2D (Table 1) [57].

With regard to specific food-derived compounds that could drive these epigenetic changes, the trimethylamine *N*-oxide (TMAO) pathway is involved in the development of IR by affecting certain epigenetic mechanisms. Trimethylamine is derived from dietary choline (meat, milk, grain, egg and their derived products, composite dishes, and fish) and l-carnitine (red meat and milk) by certain microbiota species and further oxidized to TMAO in the liver. The POUNDS Lost Trial included 510 overweight and obese individuals exposed to a low-calorie diet for two years and found that a decrease in choline and l-carnitine levels was significantly related to weight loss, indicating it might serve as a predictive marker of response to weight-loss treatment [58, 59,60]. The same study documented a significant improvement in IR associated with decreases in choline and l-carnitine [61]. Moreover, non-esterified fatty acids are known to induce IR and thus impair β-cell function [62]. To better understand the functional impact of free fatty acids on β-cell functionality, approximately 1000 islets from 13 human donors were cultured for 48 h with either palmitate or control media, and genome-wide mRNA expression and DNA methylation were assessed. Differences in expressed genes and global DNA methylation levels were found in palmitate-treated human islets, including in T2D-associated genes such as *TCF7L2, GLIS3*, *HNF1B*, and *SLC30A8* (Table 1) [63]. Finally, we previously mentioned that SCFAs including butyrate could also induce HDAC changes and thus serve as a potential epigenetic biomarker. Consistent with this notion, oral administration of gamma-aminobutyric acid (GABA)–enriched rice bran to obese rats showed an attenuation of metabolic syndrome induced by high-fat diet, which was driven by alterations in fecal SCFA levels with increased levels in butyrate and propionate and decreased levels of acetate. Also, an increase in butyrate-producing bacteria (particularly those that use acetate as a substrate, *Anaerostipes* and *Anaerostipes* spp.) was seen, which was subsequently associated with an increased release of GLP-1 [64]. Since plasma GABA levels were increased upon lean donor fecal microbiota transplantation, we postulate that specific dietary compounds can regulate metabolism via GABA derived from specific intestinal bacterial strains [65].

More specifically related to T2D, a recent study using peripheral blood mononuclear cells showed that HDAC3 activity and HDAC3 mRNA levels were positively correlated with IR [66]. Although the direct effect of other diabetes medications on the epigenome is not known, a recent intriguing publication by Zhong et al. revealed that the biguanide T2D drug metformin might affect DNA methylation. Metformin indirectly increases DNA methylation of specific tumor-promoting pathway genes and inhibits cell proliferation (Table 1) [67••].

## Dietary Effects of Epigenetic Modulation

Nutrition exerts its effect on epigenetic markers from early life. Good evidence regarding the lifelong impact of such epigenetic programming comes from the Dutch Hunger Winter Families Study. Compared with their non-famine-exposed siblings, subjects who had periconceptional exposure to famine were characterized by lower methylation of the insulin growth factor 2 (IGF2) gene 60 years later [68]. Moreover, the offspring of mothers who had been exposed to famine in utero had increased neonatal adiposity [69]. The underlying pathophysiological mechanisms might be driven by saturated fatty acid overload, which is known to induce distinct epigenetic changes in human adipose tissue. Indeed, DNA methylation can predict weight increase in response to overfeeding in humans [70]. Further evidence of a role for bacterial species in this adiposity phenotype comes from a short-term 12-week low-fat/low-calorie diet intervention in 75 patients with NASH who were treated either with probiotics or placebo. The authors showed that probiotic treatment had a significant decrease in BMI and liver stiffness in patients with NASH, with a concomitant change in the composition of gut microbiota [71].

## Conclusions

A crucial characteristic of epigenetic modification is that it is reversible and modifiable, and thus a possible target for therapeutic intervention for IR and T2D [72] either via dietary, microbiota, or pharmacological therapy-based interventions. For example, changes in microbiota or microbiota-derived metabolite production can be a way to achieve some of the desired specific epigenetic modifications and to reverse deleterious insulin-resistant obesity. Moreover, several other dietary small compounds have been shown to target proteins or genetic regulatory regions, altering epigenetic programming of organs involved in metabolism. However, a myriad of challenges must be overcome to perform high-quality research in epigenetics. Multiple factors have to be taken into consideration, such as differences between animals and humans, target tissues and cells, heterogeneity of cell composition, sample size of studies, age, sex, medication use, epigenome coverage, and interplay between the abovementioned epigenetic mechanism [11, 73]. To investigate the causality of epigenetic markers in obesity and IR/T2D, both interventional trials and long-term prospective studies on integromics (combining microbiome, epigenome, transcriptome, and plasma metabolomics) should be done in humans.
